# Phylogenetic Relationships of *Pseudorasbora*, *Pseudopungtungia*, and *Pungtungia* (Teleostei; Cypriniformes; Gobioninae) Inferred from Multiple Nuclear Gene Sequences

**DOI:** 10.1155/2013/347242

**Published:** 2013-09-10

**Authors:** Keun-Yong Kim, Myeong-Hun Ko, Huanzhang Liu, Qiongying Tang, Xianglin Chen, Jun-Ichi Miyazaki, In-Chul Bang

**Affiliations:** ^1^Department of Research and Development, NLP Co., Ltd., Busan 619-912, Republic of Korea; ^2^Department of Life Sciences & Biotechnology, Soonchunhyang University, Asan 336-745, Republic of Korea; ^3^Key Laboratory of Aquatic Biodiversity and Conservation, Institute of Hydrobiology, Chinese Academy of Sciences, Wuhan 430072, China; ^4^School of Life Science, South China Normal University, Guangzhou 510631, China; ^5^Faculty of Education and Human Sciences, University of Yamanashi, Yamanashi 400-8510, Japan

## Abstract

Gobionine species belonging to the genera *Pseudorasbora*, *Pseudopungtungia*, and *Pungtungia* (Teleostei; Cypriniformes; Cyprinidae) have been heavily studied because of problems on taxonomy, threats of extinction, invasion, and human health. Nucleotide sequences of three nuclear genes, that is, recombination activating protein gene 1 (*rag1*), recombination activating gene 2 (*rag2*), and early growth response 1 gene (*egr1*), from *Pseudorasbora*, *Pseudopungtungia*, and *Pungtungia* species residing in China, Japan, and Korea, were analyzed to elucidate their intergeneric and interspecific phylogenetic relationships. In the phylogenetic tree inferred from their multiple gene sequences, *Pseudorasbora*, *Pseudopungtungia* and *Pungtungia* species ramified into three phylogenetically distinct clades; the “*tenuicorpa*” clade composed of *Pseudopungtungia tenuicorpa*, the “*parva*” clade composed of all *Pseudorasbora* species/subspecies, and the “*herzi*” clade composed of *Pseudopungtungia nigra*, and *Pungtungia herzi*. The genus *Pseudorasbora* was recovered as monophyletic, while the genus *Pseudopungtungia* was recovered as polyphyletic. Our phylogenetic result implies the unstable taxonomic status of the genus *Pseudopungtungia*.

## 1. Introduction

Species of the subfamily Gobioninae (or gudgeons) (Teleostei; Cypriniformes; Cyprinidae) are mostly distributed in East Asia [1–4] except several species belonging to the genera *Gobio* and *Romanogobio* in Europe [5]. Among them, the slender topmouth gudgeon *Pseudorasbora elongata* endemic to China faces a high risk of extinction due to habitat degradation and loss and fishing [6]. *Pseudorasbora interrupta* was recently erected as novel species [7]. The topmouth gudgeon (or the stone morocco) *Pseudorasbora parva*, which was first reported from Nagasaki, Japan, is widely distributed in East Asia [1] and has rapidly extended its habitats either naturally or artificially to all of Europe and parts of North Africa during the last 50 years [8, 9]. Moreover, this freshwater fish is notorious as the second intermediate host of the liver fluke *Clonorchis sinensis* [10] and is a carrier of the rosette agent (*Sphaerothecum destruens*) which inhibits spawning and causes increased mortality in native European fish species [11]. *Pseudorasbora pumila pumila* and *Pseudorasbora pumila* subsp. originally inhabited limited areas in northern and middle parts on Honshu of Japan, respectively, but their distributions have been further restricted to patchily discrete locations due to loss of their habitats and invasion of *Pseudorasbora parva* into their habitats. Thus, the Japanese Ministry of the Environment designated them as critically endangered subspecies. The striped shiner *Pungtungia herzi *Herzenstein, which was first reported from Chungju, Korea [1, 4, 12], resides in China, Japan, and Korea [1]. The black shiner *Pseudopungtungia nigra* endemic to Korea was reported as a novel genus and species by Mori [13]. This species was reported to inhabit the Geum River, the Mangyeong River, and the Ungcheon Stream in Korea [14, 15] but believed to be regionally extinct in the latter due to water impoundment and pollution [4, 16]. Because of the threat of extinction, it was designated as an endangered species in 1997 by the Ministry of Environment of Korea and protected by national legislation. The slender shiner *Pseudopungtungia tenuicorpa * endemic to Korea, inhabits the upper reaches of the Han and Imjin Rivers [4, 16]. This fish species was also designated as an endangered species since 2005 due to deterioration of its natural habitats.

Despite great concerns on conservation the taxonomic positions of *Pseudorasbora elongata*, *Pseudopungtungia nigra,* and *Pseudopungtungia tenuicorpa* are still unsettled. For example, it was interesting that *Pseudorasbora elongata* showed closer phylogenetic affiliation to *Pungtungia herzi* rather than congeneric species based on the mitochondrially encoded cytochrome *b* gene (*mt-cyb*) sequences [17]. Meanwhile, Kang [18] suggested transferring two *Pseudopungtungia *species to the genus *Pungtungia *based on synapomorphic osteological characters such as jaws supporting the form of mouth, suspensorial elements, and hyoid arch, but they still remain in the former genus [4].

Despite problems with taxonomy, threats of extinction, invasion, and human health, there are not enough molecular data for *Pseudorasbora*, *Pseudopungtungia,* and *Pungtungia* species to provide compelling answers to questions about their intergeneric and interspecific phylogenetic relationships (e.g., Yang et al. [17]), genetic variation for conservation (e.g., Konishi and Takata [19]), phylogeography (e.g., Watanabe et al. [20]), divergence time estimation (e.g., Liu et al. [21]), and developing monitoring markers for tracing their dispersal route. The genetic data available for those species up to date are mostly composed of nucleotide sequences from a single mitochondrially encoded gene: the *mt-cyb*, which is maternally inherited and thus provides insufficient evidence for resolving their phylogenetic relationships.

Recently, phylogenetic markers of nuclear genes were deciphered and successfully applied for reconstructing phylogenetic trees across diverse Cypriniform species [22–25]. In this study, we analyzed multiple nuclear gene sequences of eight species and subspecies of *Pseudorasbora*, *Pseudopungtungia,* and *Pungtungia* residing in China, Japan, and Korea to elucidate their molecular phylogenetic relationships.

## 2. Materials and Methods

### 2.1. Specimen and Genomic DNA Extraction

Fish specimens used in this study were captured with a spoon net (mesh size: 4 × 4 mm) from river drainages of China, Japan, and Korea. The specimens we used were transported to the laboratory alive and killed rapidly with formaldehyde after anaesthetizing them by submerging into a solution containing a fish anaesthetic agent, Tricaine Methane Sulphonate (MS222) (Aqualife TMS, Syndel Laboratories, Ltd., Canada). The specimens were deposited in the fish collection of Soonchunhyang University (SUC; Asan, Republic of Korea), Chinese Academy of Sciences (Wuhan, China), and South China Normal University (Guangzhou, China). Their detailed sampling information was provided in Table 1.

A small piece of a pectoral or anal fin tissue was excised from each specimen to extract genomic DNA (gDNA). It was incubated in 500 *μ*L of TNES-Urea buffer (10 mM Tris-HCl, pH 8.0; 125 mM NaCl; 10 mM EDTA, pH 8.0; 1% SDS; 6 M urea; [26]) containing 100 *μ*g of proteinase K (Sigma-Aldrich, St. Louis, MO, USA) at 37°C for a week, followed by separation with phenol : chloroform : isoamyl alcohol (25 : 24 : 1) solution and ethanol precipitation. The extracted gDNA was finally resuspended in 50 *μ*L of TE buffer (10 mM Tris-HCl, pH 8.0; 1 mM EDTA, pH 8.0). Its quantity and quality were checked using a spectrophotometer, NanoDrop 1000 (Thermo Fisher Scientific, Wilmington, DE, USA) and by electrophoresis in a 0.7% agarose gel after staining with GelRed Nucleic Acid Gel Stain (Biotium, Hayward, CA, USA).

### 2.2. PCR Amplification and Sequencing

For phylogenetic analysis, three nuclear genes, that is, recombination activating gene 1 (*rag1*), recombination activating gene 2 (*rag2*), and early growth response 1 gene (*egr1*), were selected based on previous studies [23, 24]. Information for the primers used in this study is shown in Table 2. PCR reactions were carried out in a 20 *μ*L reaction volume using *AccuPower* PCR Premix (Bioneer, Daejeon, Republic of Korea), including 50 ng of gDNA and 0.2 *μ*M of forward and reverse primers.

PCR was run with the following thermal cycling profile in a DNA Engine DYAD Peltier Thermal Cycler (MJ Research Inc., Waltham, MA, USA): an initial denaturation at 94°C for 3 min, 25–35 cycles of denaturation at 94°C for 30 s, annealing at 50–52°C for 30 s, and elongation at 72°C for 1 min. The reaction was completed with a final elongation at 72°C for 7 min. The PCR product was purified with the *AccuPrep* PCR Purification Kit (Bioneer). After cycle sequencing with the ABI PRISM BigDye Terminator v3.1 Cycle Sequencing Ready Reaction Kit (Applied Biosystems Inc., Foster City, CA, USA), the purified product was directly sequenced on an ABI 3730xl DNA Analyzer (Applied Biosystems Inc.) with PCR primers by a commercial company, Macrogen Inc. (Seoul, Republic of Korea). Electropherograms were assembled in BioEdit 7.0.5 [28] and corrected manually. The sequences analyzed in this study were deposited in GenBank (http://www.ncbi.nlm.nih.gov/genbank/) under accession numbers KF468594-KF468626 (Table 1).

### 2.3. Phylogenetic Analyses

Nucleotide sequences of the *rag1*, *rag2,* and *egr1* genes of eight *Pseudorasbora*, *Pseudopungtungia,* and *Pungtungia* species analyzed in this study (Table 1) were aligned with ClustalW in BioEdit [28]. Two *Sarcocheilichthys *species were used as outgroups, based on previous molecular phylogenetic [17, 25, 29] and morphological [18] studies. The three nuclear genes were concatenated according to genes. There were no indels in the nucleotide matrix that consisted of 1,488, 1,120, and 1,087 bp for each gene, respectively. The nucleotide matrix is available upon request.

Maximum likelihood (ML) analysis was performed with RAxML 7.0.4 [30, 31]. The concatenated nucleotide matrix was partitioned according to genes. The RAxML search was executed for the best-scoring ML tree in one single program run (the “-f a” option) instead of the default maximum parsimony starting tree. The best-scoring ML tree of a thorough ML analysis was determined under the GTRMIX model in 200 inferences. Statistical support was evaluated with 1,000 nonparametric bootstrap inferences.

Bayesian inference (BI) analysis was carried out in MrBayes 3.1.2 [32] after partitioning the nucleotide matrix according to genes. MrModeltest 2.3 [33] in PAUP* 4.0b10 [34] was used to determine the best-fit evolutionary model by Akaike Information Criterion (AIC) for each gene and selected the SYM+Γ, K80+I, and HKY+I models, for the *rag1*, *rag2,* and *egr1* genes, respectively. All model parameters were unlinked across partitions, and all partitions were allowed to have different rates. Two independent Metropolis-coupled Markov chain Monte Carlo (MCMCMC) runs were performed with four simultaneous chains (three heated and one cold) and random starting trees for 5,000,000 generations, sampling parameters, and topologies every 100 generations. Burn-in was determined by checking the convergence of likelihood values across MCMCMC. A total of 500 out of 50,001 resulting trees were discarded as “burn-in.” The last trees after convergence were used to construct a 50% majority-rule consensus tree and to summarize posterior probability support for each node.

## 3. Results

**Figure 1:**
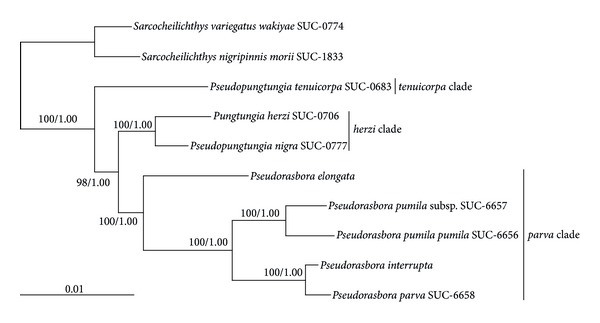
Maximum likelihood (ML) trees of gobionine species belonging to the genera *Pseudorasbora*, *Pseudopungtungia,* and *Pungtungia* inferred from multiple nuclear genes, that is, the recombination activating gene 1 (*rag1*), recombination activating gene 2 (*rag2*), and early growth response 1 gene (*egr1*). ML and Bayesian inference (BI) trees were reconstructed after partitioning the concatenated nucleotide matrix according to genes. Bootstrap values above 50% of ML analysis and posterior probabilities above 0.90 of BI analysis were shown at each branch node. The scale bar indicates substitutions/site.

ML and BI trees inferred from the multiple nuclear gene sequences generated identical tree topologies. In the phylogenetic tree, species belonging to the genera *Pseudorasbora*, *Pseudopungtungia, *and *Pungtungia* formed a monophyletic group with the highest level of confidence with respect to the *Sarcocheilichthys* outgroups (Figure 1).

In the phylogenetic tree, *Pseudorasbora*, *Pseudopungtungia, *and *Pungtungia* species were ramified into three distinct clades; the “*tenuicorpa*” clade composed of a single species, *Pseudopungtungia tenuicorpa*, the “*herzi*” clade of *Pseudorasbora nigra* and *Pungtungia herzi*, and the “*parva*” clade of *Pseudorasbora elongata*, *Pseudorasbora interrupta*, *Pseudorasbora parva* and *Pseudorasbora pumila pumila*,* Pseudorasbora pumila *subsp. (Figure 1). Among those clades, “*tenuicorpa*” clade placed at the basal position, giving rise to two ramifying “*herzi*” and “*parva*” clades, supported by 98% bootstrap value in ML tree and 1.00 posterior probability in BI tree. The “*herzi*” and “*parva*” clades were supported with the highest statistical supports. Within the “*parva*” clade, *Pseudorasbora elongata* formed the sister-group relationship to the lineage composed of *Pseudorasbora pumila pumila*,* Pseudorasbora pumila *subsp.,* Pseudorasbora interrupta,* and *Pseudorasbora parva*. The former two consistently separated from the latter two.

## 4. Discussion

In the phylogenetic tree, the genus *Pseudorasbora* was recovered as monophyletic, but the genus *Pseudopungtungia* was recovered as polyphyletic; the monotypic *Pungtungia herzi* was closely affiliated to *Pseudopungtungia nigra* with highest statistical supports, and *Pseudopungtungia tenuicorpa *was placed at the basal position among *Pseudorasbora*, *Pseudopungtungia,* and *Pungtungia* species.

Previous molecular phylogenetic studies inferred from nuclear or mitochondrial gene sequences [17, 25, 28] clearly revealed the monophyly of the genera *Pseudorasbora* and *Pungtungia*. However, those studies did not include a closely related genus (i.e., *Pseudopungtungia*) and all *Pseudorasbora* species (i.e., *Pseudorasbora interrupta *and both* Pseudorasbora pumila *subspecies). Overall tree topologies generated in this study after including all those species revealed the clear monophyletic nature of the three genera *Pseudorasbora*, *Pungtungia,* and *Pseudopungtungia* from China, Japan, and Korea with respect to *Sarcocheilichthys* outgroups. This is completely or partially congruent with previous phylogenetic assumptions based on osteology [18, 35] and anatomy (vertebral formula; [36]). Meanwhile, our phylogenetic trees recovered the genus *Pseudorasbora* as monophyletic and the genus *Pseudopungtungia* as polyphyletic. *Pseudopungtungia nigra* showed the closest phylogenetic affiliation to *Pungtungia herzi*, and *Pseudopungtungia tenuicorpa* was clearly separated not only from those two species but also from the five *Pseudorasbora* species and subspecies.

In accordance with the polyphyletic nature of the genus *Pseudopungtungia*, Kim [37] mentioned significant morphological differences between *Pseudopungtungia nigra* and *Pseudopungtungia tenuicorpa* in the body shape and crossbars in fins except the mouth shape and the unstable taxonomic status of the genus *Pseudopungtungia*. Mori [13] morphologically differentiated *Pseudopungtungia nigra* from *Pungtungia herzi* by the mouth shape and fin coloration and erected the former as a novel genus and species. However, Banarescu [38] described their similarities in the mouth shape, lips, and jaws that are congruent with our molecular phylogenetic result. The close relationship can also be explained by the occurrence of a natural hybrid between them [39]. Independently, Kim et al. [40] carried out the polyacrylamide gel electrophoresis of muscle proteins extracted from Korean gobionine species to investigate their systematic relationships. Their result revealed many similarities among species of *Coreoleuciscus*,* Pseudorasbora*, *Pseudopungtungia,* and *Pungtungia* and the close relationship between *Pseudopungtungia nigra *and *Pungtungia herzi *among them, which is also congruent with our results.

The monophyly of the genus *Pseudorasbora* reflected the current taxonomic classification, which includes *Pseudorasbora elongata*, *Pseudorasbora interrupta*, *Pseudorasbora parva*, *Pseudorasbora pumila pumila,* and *Pseudorasbora pumila *subsp. Banarescu and Nalbant [41] mentioned that *Pseudorasbora elongata* has a notably distinct taxonomic position from other *Pseudorasbora* species, because of its elongated body and snout and longitudinal blackish stripes as *Pungtungia*. This is congruent with our phylogenetic tree, because *Pseudorasbora elongata* was placed at the basal position separated from other *Pseudorasbora* species and subspecies. This is also congruent with the result of Yang et al. [17] based on the *mt-cyb* gene. However, Yang et al. [17] and Liu et al. [21] showed that *Pseudorasbora elongata* consistently clustered with *Pungtungia herzi* and clearly separated from congeneric *Pseudorasbora parva* and *Pseudorasbora pumila* in their *mt-cyb* trees. Xiao et al. [7] mentioned the close relationship of *Pseudorasbora interrupta* to *Pseudorasbora parva* and *Pseudorasbora pumila*, which is congruent with our phylogenetic tree. In this study, *Pseudorasbora interrupta* has a closer phylogenetic relationship to *Pseudorasbora parva* than the two *Pseudorasbora pumila* subspecies.

Our result shows the unstable taxonomic status of the genus *Pseudopungtungia* and suggests a novel genus should be erected to accommodate *Pseudopungtungia tenuicorpa* in a future taxonomic study. Besides resolving phylogenetic relationships, the nucleotide sequence information presented in this study will provide useful baseline data for developing recovery plans of endangered species and subspecies investigated in this study (i.e., *Pseudopungtungia nigra* and *Pseudopungtungia tenuicorpa*, *Pseudorasbora elongata* and *Pseudorasbora pumila* subsp.) because clarification of their phylogenetic positions is the prerequisite for such efforts.

## Figures and Tables

**Table 1:** Sampling information of gobionine species used in the phylogenetic analyses.

Species	Voucher no.	Sampling site	Drainage	GenBank acc. no.		
				*rag1 *	*rag2 *	*egr1 *
*Pseudopungtungia nigra *	SUC-0777	Geumsan, Korea	Geum River	KF468619	KF468608	KF468597
*Pseudopungtungia tenuicorpa *	SUC-0683	Yangpyeong, Korea	Han River	KF468618	KF468607	KF468596
*Pseudorasbora e* *lo* *ng* *at* a*	—	China	—	KF468621	KF468610	KF468599
*Pseudorasbora i* *nt* *er* *ru* *pt* a*	—	China	—	KF468623	KF468612	KF468601
*Pseudorasbora parva *	SUC-6658	Kasumigaura, Ibaraki Pref., Japan	A small tributary of Lake Kasumigaura	KF468626	KF468615	KF468604
*Pseudorasbora pumila pumila *	SUC-6656	Nagano, Nagano Pref., Japan	An irrigative pond	KF468624	KF468613	KF468602
*Pseudorasbora pumila *subsp.	SUC-6657	Bred in Lake Biwa Museum, Shiga Pref., Japan	—	KF468625	KF468614	KF468603
*Pungtungia herzi *	SUC-0706	Yangpyeong, Korea	Han River	KF468620	KF468609	KF468598
*Sarcocheilichthys nigripinnis morii *	SUC-1833	Seocheon, Korea	Gilsan Stream	KF468617	KF468606	KF468595
*Sarcocheilichthys variegatus wakiyae *	SUC-0774	Geumsan, Korea	Geum River	KF468616	KF468605	KF468594

*Detailed information is not provided by the authors for protecting their natural habitats.

**Table 2:** Information of PCR primers used in this study.

Gene	Primer	Sequence (5′ → 3′)	Reference
Recombination activating gene 1 (*rag1*)	RAG1-1495f3	CAGTAYCAYAAGATGTACCG	Kim and Bang [27]
	RAG1-3067r	TTGTGAGCYTCCATRAACTT	Kim and Bang [27]
Recombination activating gene 2 (*rag2*)	RAG2-108f	CCVARACGCTCATGTCCAAC	This study
	RAG2-1324r	TGGARCAGWAGATCATKGC	This study
Early growth response 1 gene (*egr1*)	EGR1-291f	CACAGGMCGTTTCACCCTYG	Modified from Chen et al. [24]
	EGR1-1456r	GACAGGRGARCTGTAGATGTT	Modified from Chen et al. [24]
